# Mechanical Performance and Precipitation Behavior in Al-Si-Cu-Mg Cast Alloys: Effect of Prolonged Thermal Exposure

**DOI:** 10.3390/ma15082830

**Published:** 2022-04-12

**Authors:** Mohamed H. Abdelaziz, Agnes M. Samuel, Herbert W. Doty, Victor Songmene, Fawzy H. Samuel

**Affiliations:** 1 Département PEC, Université Française d’Égypte, Ville Shorouk, Cairo 11837, Egypt; mohamed.abdelaziz@ufe.edu.eg; 2 Département des Sciences Appliquées, Université du Québec à Chicoutimi, Chicoutimi, Saguenay, QC G7H 2B1, Canada; fawzy-hosny.samuel@etsmtl.ca; 3 General Motors Global Technology Center, Warren, MI 48093-2350, USA; herb.doty@gm.com; 4 Département de Génie Mécanique, École de Technologie Supérieure, Montreal, QC H3C 1K3, Canada; victor.songmene@etsmtl.ca

**Keywords:** Al-Si-Cu-Mg alloy, thermal exposure, heat treatment, transition element additions, mechanical properties, microstructure, fractography

## Abstract

Al-Si-Cu-Mg cast (354) alloys are used in the automotive sector owing to their remarkable properties which are achievable after applying appropriate thermal treatments. Zirconium, Nickel, and Manganese were added to this category of Al-alloys to preserve good mechanical properties while being exposed to elevated temperatures for long times. The ultimate and yield strength values obtained at room temperature for the stabilized (thermally-exposed) T5-treated condition were comparable to those of the stabilized T6-treated condition, whereas the same properties for T5-treated alloys were higher than those of T6-treated ones for elevated-temperature tensile testing. Interestingly, the results showed that the addition of 0.75 wt.% Mn was competitive with the addition of 2 and 4 wt.% Ni with respect to the elevated-temperature and ambient temperature strength values, respectively. In addition, the Mn-containing alloy M3S exhibited improved ductility values at ambient temperature and at 250 °C, compared to the Ni-containing alloys. Examination of the fracture surface of tested samples revealed the advantageous role of sludge particles in enhancing the performance of Mn-containing alloys through their resistance to the propagation of cracks that developed in many intermetallic phases. This finding is considered to be economically significant in view of the lower price of manganese compared to that of nickel.

## 1. Introduction

Heat-treatable aluminum alloys play an important role in lowering vehicle weight and promoting fuel economy in the automotive industry because of their lightweight and improved properties, such as outstanding response to plastic deformation and casting processes, good corrosion resistance, and high strength/weight ratio. The Ducker Worldwide Company conducted a survey on the amount of aluminum alloys used in North American automotive vehicles; the results showed that aluminum usage has increased from about 37 kg in 1974 to 155 kg in 2012 and it is projected to reach 250 kg by 2025 [1]. On the technological side, some concerns regarding the use of aluminum alloys in the automotive sector have been raised regarding the reliability of aluminum engine components when employed in modern engines with higher specific powers which produce increased combustion pressure and temperature [2,3,4]. The replacement of iron-based alloys in engine components by others made of lighter aluminum alloys should not impair the performance of the vehicles, so that all efforts towards enhancing the properties of aluminum alloys to meet service requirements will be beneficial towards reducing emissions and developing greener societies.

It is well known that the performance of heat-treatable aluminum alloys deteriorates seriously when subjected to elevated temperatures, especially when this temperature exceeds the aging temperature (for T6-heat treated alloys), as a result of alloy softening; this deterioration in the mechanical properties is mainly dependent on both the exposure temperature and duration. Recently, several studies [5,6,7,8,9,10,11,12] have been undertaken in an attempt to overcome the problems encountered when utilizing aluminum alloys in high temperature applications. The hypothesis is to form more stable intermetallic compounds and precipitates which are coarsening resistant at elevated temperatures. The common approach was to use minor additions of transition elements such as Zr, Sc, Ni, Ti, V, and Mn to cast aluminum alloys to form more stable intermetallic compounds.

Ceschini et al. [13] compared the capabilities of T6-treated A356 and C355 alloys after being exposed to high temperature (210 °C) for 41 h. The results showed the superiority of C355 alloy (containing Cu) over the A356 alloy (Cu-free) in the tensile properties in the overaged condition. However, the variation in the tensile properties of the two alloys in the T6 condition was not that high. Feng et al. [14] investigated the microstructure evolution and the mechanical properties of Al-Si-Cu-Mg-Ni alloy following its exposure to 350°C for times of up to 1000 h. The results showed that in the first 100 h, the ultimate tensile strength at both room and elevated temperature decreased remarkably, as well as the Brinell hardness. After the first 100 h stabilization time and up to 1000 h, the deterioration in the mechanical properties was not significant. The authors also found that the eutectic silicon particles grew continuously with the increase in exposure time as well as the amount of Q phase. Jabra et al. [15] examined six aluminum alloys which were exposed to different high temperatures (80°, 230°, and 290 °C) for different holding times (0.1, 0.5, 2, 10, 100, and 1000 h). They found that the strength of the alloys deteriorated considerably with higher temperatures and longer exposure times, vice-versa for their ductility. In a TEM study, Kai et al. [16] found that the strength of 7050 Al alloy was reduced when the alloy was exposed to elevated temperature for longer periods; the amount of reduction in strength was proportional to the temperature. The thermal exposure was done at different temperatures (100°, 125°, and 150 °C) for 500 h. The authors explained this reduction in strength as a result of the coarsening behavior of both GP zones and ƞ’ precipitates, and also due to the increasing width of the precipitate free zones (PFZs).

The quality index concept was first developed by Drouzy et al. [17] to analyze their tensile data on Al-7Si-Mg alloys. Their ideas were further developed by Cáceres [18] to include other Al alloy systems. As quality charts will be used to analyze the tensile data obtained in the present study, the development of the quality index as given by Drouzy et al. [17] and Cáceres [18] is provided in detail in the Appendix A at the end of this article [19,20,21,22,23,24,25,26,27,28,29,30,31].

The current study is expected to provide in-depth understanding and knowledge of the effect of prolonged thermal exposure at elevated temperature on the room- and elevated-temperature mechanical properties of the alloys studied; the evolution of the strengthening precipitates and the fracture surfaces following prolonged thermal exposure are also examined. In the Appendix A section, the quality index concept and quality charts are used to analyze and further interpret the results; the Cáceres quality concept will be used for the mechanical properties obtained at room temperature, whereas Drouzy’s concept will be used with elevated-temperature properties.

## 2. Materials and Methods

### 2.1. Preparation of Alloys

A set of chemically modified Al-Si-Cu-Mg (354-type) alloys were investigated in this study. The reference alloy, which is coded M1S, is 200 ppm Sr-modified Al-Si-Cu-Mg with ~0.3 wt.% Zr addition. This alloy was selected as the reference alloy owing to its enhanced mechanical behavior, which has been previously reported in other investigations by the same research group [5,32]. Other alloys under investigation were developed by incorporating selective additions of Nickel (Ni) and Manganese (Mn) to the reference alloy. Chemical composition and codes of the alloys investigated are provided in Table 1.

An electrical resistance furnace was used to melt the charge of 354 alloy ingots. Due to the presence of Zr- and Ni-containing compounds originating from the master alloys, the melt was superheated at 800 °C ± 5 °C in order to make sure that all compounds were efficiently dissolved. To adjust the chemistry of the alloys, Si, Cu, and Mg were added in the form of pure elements, whereas Zr, Ni, and Mn were added in the form of Al-15%Zr, Al-20%Ni, and Al-25%Mn master alloys, respectively. The melt was stirred, degassed, and skimmed before casting in a preheated (450 °C) ASTM B-108 mold through a preheated pouring cup with a ceramic foam filter (15 ppi); this mold was used to prepare standard tensile bars with a diameter of 12.7 mm. For hardness test samples, an L-shaped mold was used to produce coupons with a geometry of 35 mm × 30 mm × 80 mm.

The five alloys were heat treated according to standard procedures of T5 and T6 tempers, as detailed in Table 2. Heat treated samples were then stabilized at 250 °C for 100 and 200 h in order to investigate the effect of such stabilization treatment on ambient- and elevated-temperature mechanical performance. 

### 2.2. Mechanical Testing

A MTS Servohydraulic mechanical testing machine (MTS Systems Corporation, Eden Prairie, MN, USA) was used to determine the tensile properties at room temperature. A strain rate of 4 × 10^−4^ s^−1^ was employed for all the experiments. For elevated-temperature tensile properties at 250 °C, an Instron Universal mechanical testing machine (INSTRON®, Norwood, MA, USA) was used to carry out testing at a strain rate of 4 × 10^−4^ s^−1^. The testing was done at 250 °C after holding the test bar for 15 min at the testing temperature in order to homogenize the temperature of the sample to 250 °C throughout. The test sample was kept unmounted from one side inside the heating chamber during the holding process to avoid compressive stresses that might arise from the expansion of the bar, and then it was mounted from the other side and kept at the testing temperature for another 15 min. Five test bars for each alloy/condition were tested and the average values of UTS, YS, and %EL were reported. Hardness testing was carried out using a Rockwell hardness tester and F scale using a 1/16-inch steel ball indenter and a load of 60 Kgf. Ten measurements were made per sample, and the average value was reported as the Rockwell hardness value of that alloy sample/condition.

### 2.3. Advanced Microscopy Investigations

A Hitachi-SU8000 field emission scanning electron microscope (FESEM) (Hitachi-High Technologies Corporation, Tokyo, Japan) equipped with an energy dispersive X-ray spectrometer (EDS), a FEI Tecnai™ G2 F20 electron microscope equipped with an advanced control system which permits the integration of an EDAX™ chemical analysis system, as well as scanning transmission electron microscopy (STEM) (FEI Company, Hillsboro, OR, USA), and electron energy loss spectroscopy (EELS) were used to investigate the evolution of the strengthening precipitates during the prolonged thermal exposure of the heat-treated alloys.

## 3. Results and Discussion

### 3.1. Microstructural Characterization

**Figure 1:**
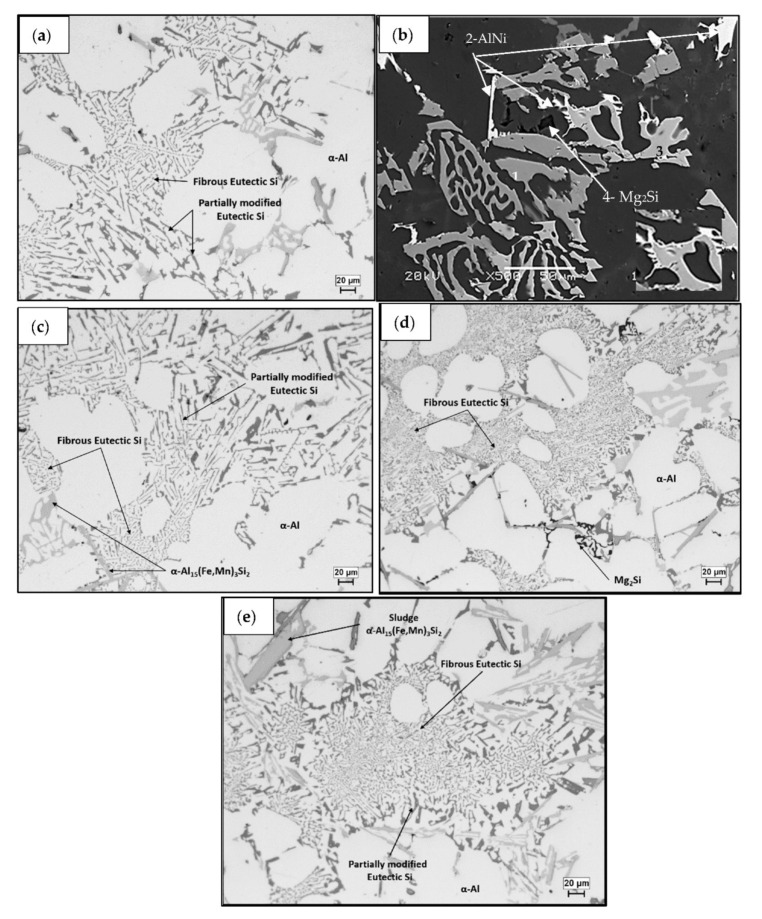
Optical microstructures of: (**a**) M1S alloy, (**b**) backscattered electron image of M2S alloy showing a mixture of 1- AlFeNi, 2-AlNi, 3-AlCuNi, and 4-Mg_2_Si phases. Note the precipitation of AlNi phase particles on the edges of AlCuNi particles in the inset micrograph (**c**) M3S alloy, (**d**) M4S alloy, (**e**) M5S alloy.

Figure 1 shows the microstructures for the alloys studied. There are a number of common microstructural features that exist in all alloys, such as α-Al dendrites, partially modified eutectic silicon particles, as well as areas of fully modified silicon particles, as shown in Figure 1a for alloy M1S. In addition, other phases were identified based on the alloy chemistry, such as Al_2_Cu, Mg_2_Si, Al_3_Zr, and α-Al_15_(Fe,Mn)_3_Si_2_.

Figure 1b displays a backscattered electron image of alloy M2S (containing 2 wt.% Ni). In addition to the common microstructural features, the image shows a mixture of AlFeNi, AlNi, AlCuNi, and Mg_2_Si phases. The AlFeNi particles are observed to exist in both script-like and platelet form. Note the precipitation of AlNi phase particles on the edges of AlCuNi particles in the inset micrograph at the bottom right of Figure 1b. In alloy M4S (containing 4 wt.% Ni), the same phases were also observed as those for alloy M2S, as well as eutectic Al-Al_3_Ni due to the high Ni content.

The presence of 0.75 wt% Mn in alloy M3S leads to precipitation of iron, preferably in the form of the α-Al_15_(Fe,Mn)_3_Si_2_ phase which is less detrimental to the mechanical properties than the needle-like β-Al_5_FeSi phase, as seen in Figure 1c. The α-Al_15_(Fe,Mn)_3_Si_2_ phase appeared in two different forms, either script-like or as polygonal particles of sludge, as observed in Figure 1e for the M5S alloy which also contains 0.75 wt% Mn.

### 3.2. Ambient-Temperature Tensile Properties

**Figure 2:**
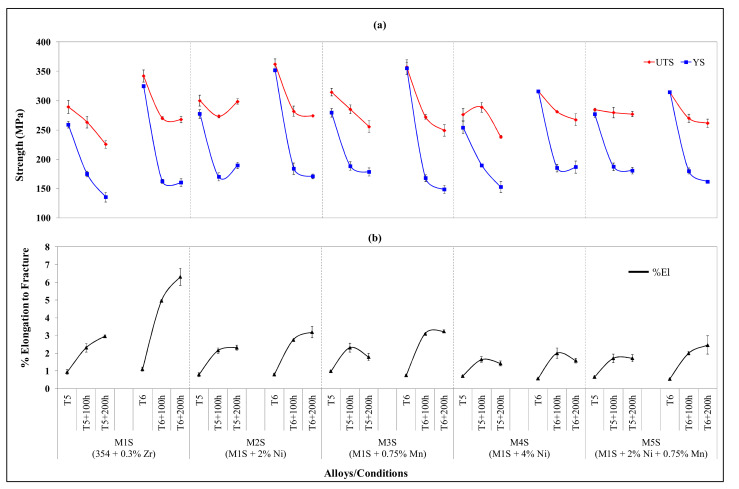
Variation in average UTS, YS, and %El values for alloys M1S through M5S in the T5, T6, and after static stabilization at 250 °C for 100 h, and 200 h (testing at ambient temperature: (**a**) UTS and YS; (**b**) %El.

Figure 2 demonstrates ambient-temperature tensile properties obtained for the alloys M1S through M5S in the T5- and T6-treated conditions, before and after thermal exposure (stabilization) at 250 °C for 100 h and 200 h. It is obvious that the thermal exposure has a serious deleterious effect on strength values, in particular, the yield strength, contrary to the ductility values which have markedly increased following exposing at 250 °C for prolonged times up to 200 h. The variation trends in the strength values (UTS and YS) of stabilized T6-treated conditions are considered consistent for the alloys studied. The stabilized T5-treated conditions, on the other hand, show irregular variation trends in relation to the strength values of the alloys studied. However, with respect to the ductility values, stabilized T5- and T6-treated conditions demonstrate similar trends in relation to the improvement in the ductility values of the investigated alloys, as can be inferred from Figure 2.

In addition, Figure 2 reveals that the strength values (UTS and YS) obtained for the stabilized T5-treated conditions are comparable to, and in various alloys exceed, those obtained for the stabilized T6-treated conditions, whereas the ductility values for stabilized T6-treated conditions are higher than those obtained with stabilized T5-treated conditions. If the observations regarding the strength values obtained after stabilization treatment are sufficiently verified, and acceptable high strength and ductility values of T5-treated conditions are attainable, this will be of great economic benefit in terms of heat treatment costs and higher production rates, because solution heat treatment, which is both time and energy consuming, is not a step used in the T5-temper process.

The improved strength values of stabilized T5-treated conditions can be attributed to the limited amount of strengthening precipitates which exist in the structure of T5-treated alloys as a result of the direct artificial aging of as-cast structures without solutionizing. Alloy softening is mainly driven by coarsening of the strengthening precipitates. Thus, by increasing the volume fraction of the coarsened precipitates, the softening behavior will be noticeable, as in the case of stabilized T6-treated alloys, while the coarsening of a limited volume fraction of precipitates will not degrade the strength values much, as is the case for the stabilized T5-treated conditions. This interpretation may be rephrased in terms of microstructural stability: the more stable the microstructure is while being exposed at an elevated temperature, i.e., when microstructural changes are kept to a minimum, the less the degradation is in the mechanical properties. This concept is better understood in terms of the ductility values shown in (Figure 2b). The ductility values in the stabilized T5-treated conditions do not change considerably compared to the T5-treated condition, whereas the opposite is apparent for the T6-treated conditions before and after stabilization.

The highest resistance to softening is associated with alloys M4S and M5S in stabilized T5- and T6-treated conditions. The overall strength values of these alloys are the lowest in the T5- and T6-treated conditions compared to the other three alloys (M1S, M2S, and M3S). In terms of absolute strength values, however, the T6-treated M2S and M4S alloys, with 2 and 4 wt.% Ni, respectively, show the best strength values after 200 h of stabilization at 250 °C. This observation can be ascribed to the mutual existence of Al-Cu-Ni and Al_3_Ni phases in all Ni-containing alloys M2S, M4S, and M5S. In particular, alloy M4S show the best resistance to softening and highest strength values after stabilization of the T6-treated alloy for 200 h, owing likely to the uniformly distributed eutectic Al-Al_3_Ni structure, which is a stable structure and therefore advantageous to the mechanical properties [33].

### 3.3. Hardness Values

**Figure 3:**
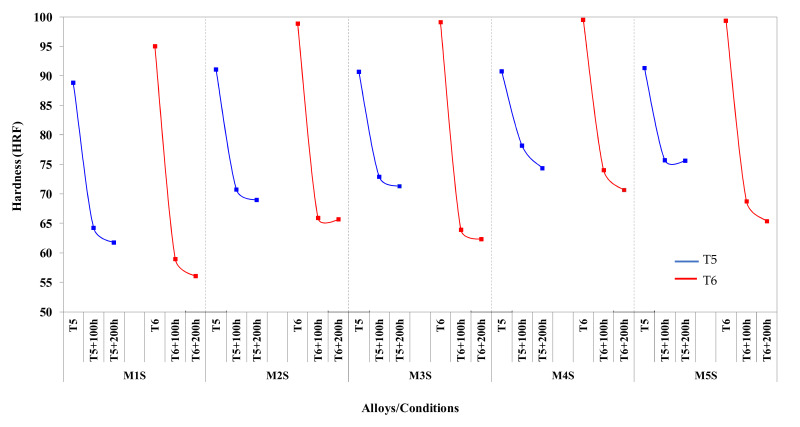
Variation in average Rockwell hardness values for alloys M1S through M5S in the T5 and T6 conditions, and after static stabilization at 250 °C for 100 h and 200 h (testing at ambient temperature).

Figure 3 displays the variations in hardness values with respect to alloy composition, applied heat treatment, and stabilization time. There is a serious drop in the hardness values following stabilization at 250 °C for 100 h of the T5- and T6-treated alloys. For example, for the T5-treated base alloy, the hardness drops from 88 HRF to 63.5 HRF and from 93.5 HRF to 61.7 HRF for the T6-treated alloy. However, further stabilization at 250 °C reduces the hardness values at a much slower rate. 

From Figure 3, it is obvious that the hardness values, before and after the stabilization treatment, are dependent on the volume fraction of intermetallic compounds present in the alloy (see Table 3). The base alloy M1S, with the lowest volume fraction of intermetallic phases, exhibits the lowest hardness values in all the conditions studied. Alloy M4S, which contains 4 wt.% Ni and has the highest volume fraction of intermetallic compounds, shows the highest hardness values for almost all of the conditions studied (cf. 93.5 with 99.5 HRF and 55.9 with 69.8 HRF for the two alloys in the T6 and T6 + 200 h/250 °C conditions, respectively). 

Hardness values of T6-treated alloys are noticeably higher than those of T5-treated alloys before the stabilization treatment. In contrast, the stabilized T5-treated conditions of 100 and 200 h at 250 °C show improved hardness values compared to those obtained with stabilized T6-treated conditions per alloy. Similar observations regarding the enhanced tensile properties of stabilized T5-treated conditions over those of stabilized T6-treated conditions were reported in the preceding subsection. This behavior can be ascribed to the fact that after stabilization, the T6-treated alloys will contain a considerable number of coarsened precipitates, which will obviously deteriorate the hardness and strength values. 

Microstructures of alloys in the T6-treated (peak-aged) condition already contain a high number of fine precipitates following solutionizing, quenching, and artificial ageing treatments. Thus, further exposure to elevated temperatures will lead to the coarsening of these fine precipitates, and hence lower their numbers and reduce their strengthening effect. Microstructures of alloys in the T5-treated condition, on the other hand, contain lower fractions of the fine precipitates, because of the artificial aging of the as-cast microstructure without solution treatment and quenching. This is because the precipitation process in the T5-temper depends on the already dissolved Cu and/or Mg in the α-Al matrix during solidification of the cast material at a high cooling rate. Accordingly, stabilization of T5-treated microstructures will not result in a rapid coarsening of the fine precipitates due to the fewer numbers of precipitates and the relatively large distances between these particles. 

Coarsening (Ostwald ripening) of the strengthening precipitates is mainly favored at elevated temperatures, i.e., during the stabilization process, where larger particles may grow further at the expense of smaller precipitate particles. This process occurs by the diffusion of atoms from the smaller particles towards the larger precipitate particles since the latter are preferred from the energy point of view. Consequently, coarsening is accompanied by a reduction in the total number of precipitates. As the coarsening phenomenon is a dissolution- and diffusion-controlled process, thus, if the particles are separated, due to their fewer numbers, by long distances as in the case of T5-treated alloys, the coarsening rate will be slower and hence the deterioration rate in hardness and strength values will be lower compared to that in the T6-treated alloys.

### 3.4. Elevated-Temperature Tensile Properties

**Figure 4:**
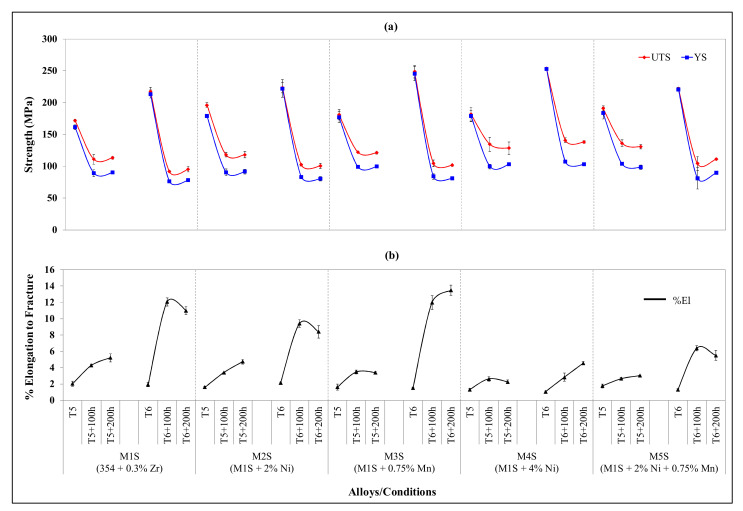
Variation in average UTS, YS, and %El values for alloys M1S through M5S in the T5 and T6, and after stabilization at 250 °C for 100 h and 200 h (testing at 250 °C). (**a**) UTS and YS; (**b**) %El.

Figure 4 presents the tensile properties of the investigated alloys obtained at 250 °C for the stabilized T5- and T6-treated conditions. An immediate observation from Figure 4 is that the stabilized T5-treated conditions exhibit better strength values (UTS and YS) than those obtained with stabilized T6-treated conditions for each alloy, except for the 4 wt.% Ni-containing M4S alloy. In this alloy, the strength values remain more or less unchanged for the stabilized T5- and T6-treated conditions. The ductility values obtained after stabilization of T5-treated conditions are dramatically lower than those obtained after stabilization of T6-treated conditions (cf. 2.26% and 4.57%); without stabilization treatment, the ductility values in the two cases differ by about 0.3% in favor of the T6-treated condition. It is also seen that alloys M4S and M5S are the least ductile when tested at 250 °C after being exposed to 250 °C for 100 and 200 h. This behavior highlights the effective resistance to softening of these alloys, following the addition of 4 wt.% Ni in alloy M4S and the combined addition of 2 wt.% Ni and 0.75 wt.% Mn in alloy M5S. Moreover, these two alloys exhibit the highest ultimate tensile and yield strengths in the case of T6-treated conditions after stabilization treatment at 250 °C for 200 h, as can be inferred from Figure 4a, which emphasizes again the effective role of the additions to these alloys in resisting softening when exposed to elevated temperatures. 

According to investigations by Rana et al. [34] and Hanafee [35], the highest benefits from Ni addition are attained when the microstructure comprises a large volume fraction with an advantageous distribution of the Al_3_Ni phase. This observation was noted for the microstructure of alloy M4S. Consequently, the addition of 4 wt.% Ni to the base alloy to form alloy M4S gives the best strength values at 250 °C after holding at the testing temperature, i.e., stabilization, for 200 h. In practice, the addition of Ni to Al-alloys is kept to a minimum because of its high price and its negative effect on ductility. In this context, it should be mentioned that while the addition of 4 wt% Ni to cast Al-alloys is neither practical nor industrially feasible, the 4 wt.% Ni-containing alloy was considered in these investigations for the sake of comparison with the other additions, i.e., 2 wt.% Ni, 0.75 wt.% Mn, and 2 wt.% Ni + 0.75 wt.% Mn. In terms of elevated-temperature tensile properties before and after the stabilization treatment, it was surprisingly found that the addition of 0.75 wt.% Mn in alloy M3S is competitive with the addition of 2 wt.% Ni in alloys M2S and M5S in regard to the strength values, and better, with respect to ductility values, as depicted in Figure 4. This would prove valuable to industry due to its economic implications. 

The enhanced ductility values of alloy M3S can be attributed to the morphological transformation of the β-Al_5_FeSi phase needles into the more compact, less detrimental α-Al_15_(Mn,Fe)_3_Si_2_ phase owing to the addition of Mn [33,36,37,38]. Increasing the manganese content over 0.5 wt.%, which is the case in the present study, will not only transform the β-Al_5_FeSi phase into α-Al_15_(Mn,Fe)_3_Si_2_ phase, but may also produce fine dispersoids capable of enhancing the mechanical performance. These fine dispersoids, which appear in the form of Al_6_Mn, are incoherent with the α-Al matrix, and hence increase the strength by hindering dislocation glide through their pinning action on dislocations. Contrastingly, the enhanced ductility arises from the change in the slip system to cross-slip due to the hindered dislocations; this cross-slip allows us to maintain good ductility of the alloy together with the increased strength values [39,40]. 

### 3.5. Precipitate Evolution during Thermal Stabilization 

**Figure 5:**
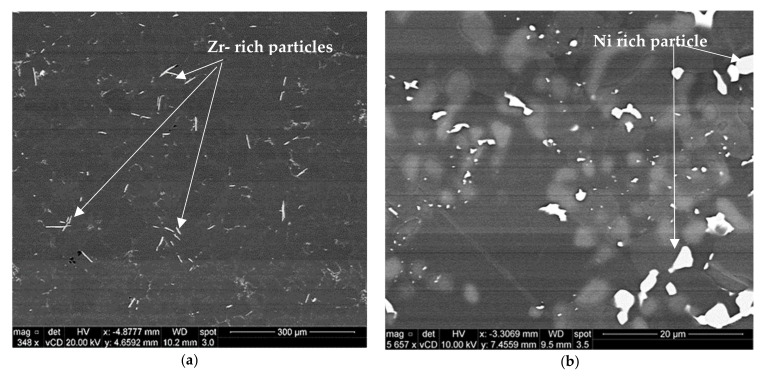

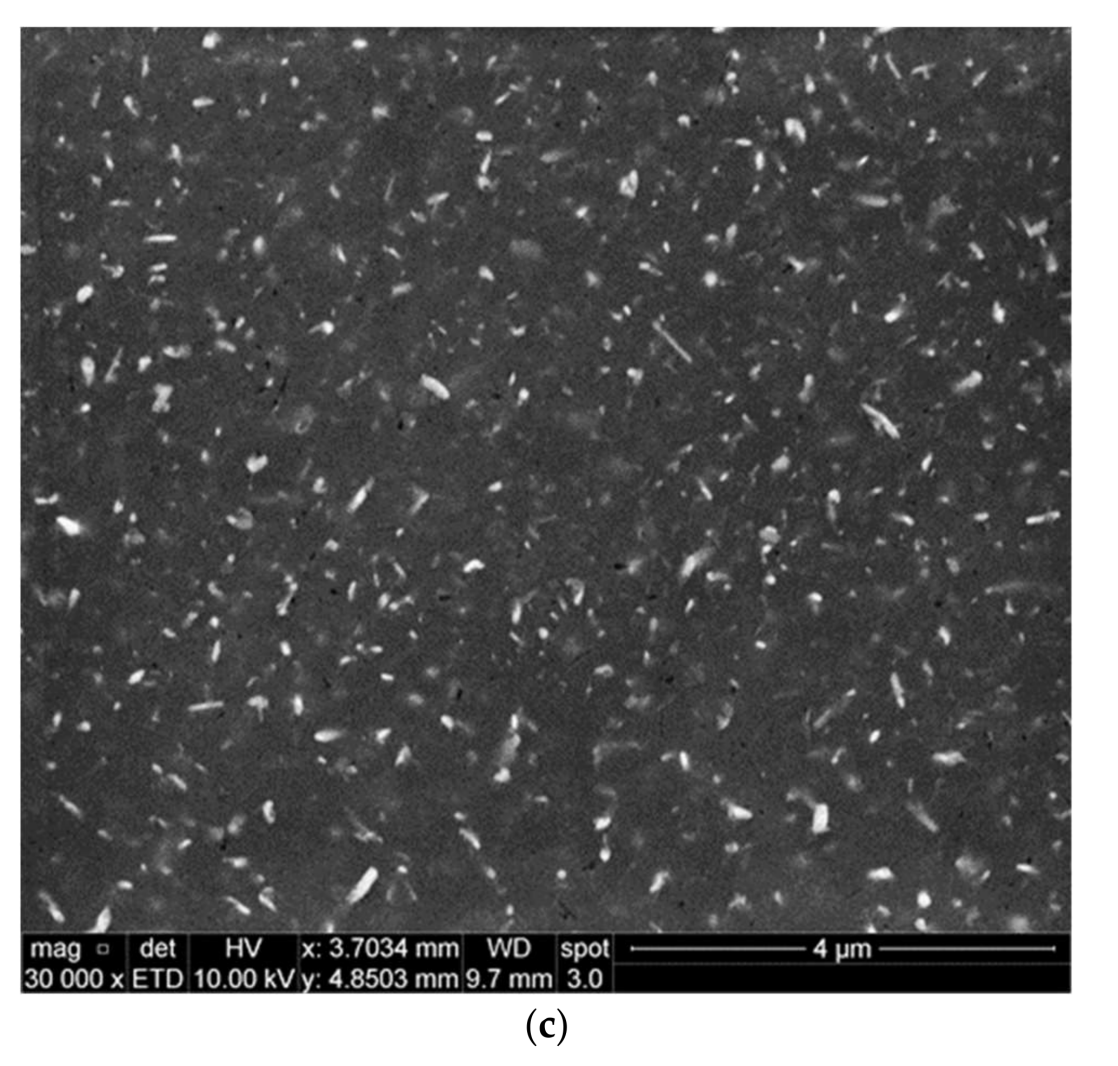
Backscattered electron images showing the size and distribution of precipitates in T6-treated alloys after stabilization at 250 °C for 1 h: (**a**) M1S, (**b**) M2S, (**c**) M3S alloys (testing at 250 °C)-mixture of Zr-, and Cu-rich phase particles.

It should be mentioned here that all electron micrographs were taken at 10 kV to be as close as possible to the examined polished surface. The characteristics and distribution of the strengthening precipitates were examined for T6-treated alloys M1S, M2S, and M3S, then stabilized at 250 °C for 1 and 200 h before testing at 250 °C. The data of alloys held at 250 °C for one hour before testing are reported as the elevated-temperature tensile properties obtained at that temperature. Low precipitation densities are observed in Figure 5 for the T6-treated M1S, M2S, and M3S alloys following one hour of stabilization at 250 °C. This may be attributed to the insufficient coarsening kinetics of the precipitates during this period. Since the coarsening behavior comprises dissolution-controlled and diffusion-controlled processes, this short time of stabilization may result only in dissolving some precipitates without completing the diffusion process.

The microstructure of alloy M3S reveals a higher number of precipitates under the same treatment/stabilization conditions in comparison to the microstructure of alloys M1S and M2S, as seen in Figure 5. This increased density of precipitates in the M3S alloy supports the possibility of the formation of the fine Al_6_Mn precipitates together with the principal strengthening precipitates θ-Al_2_Cu and S-Al_2_CuMg phases and their precursors. 

**Figure 6:**
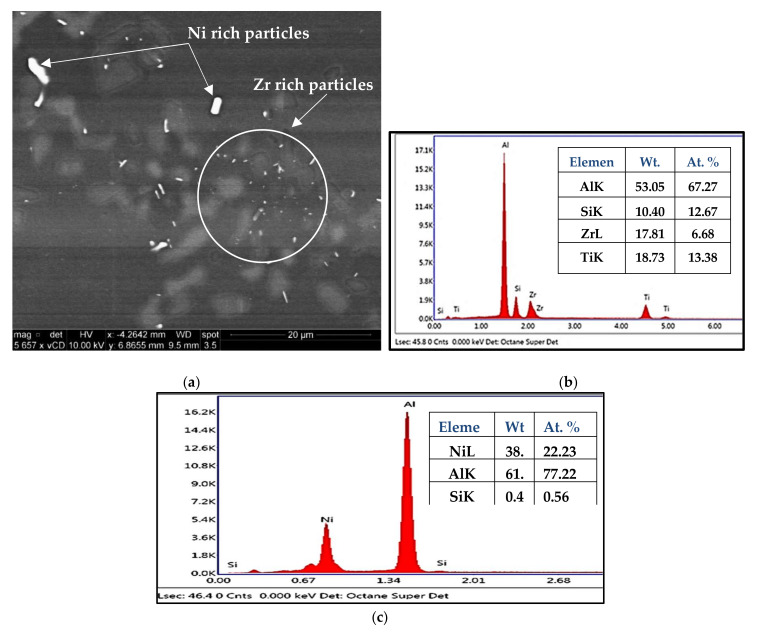
Backscattered electron images showing (**a**) bright coarse and fine dispersoids in the T6-treated M2S alloy after stabilization at 250 °C for 1 h and testing at the same temperature, (**b**) EDS spectrum corresponding to the bright particles in the circled area in (**a**), (**c**) EDS spectrum corresponding to Ni-rich particles in (**a**).

The BSE image of alloy M2S, shown in Figure 6a, reveals the presence of a certain phase whose particles exhibit different morphologies and a wide range of sizes. The associated EDS spectrum of this phase, Figure 6b, showed strong reflections of Al, Si, Zr, and Ti elements, indicating that the phase is possibly an Al_x_(Zr,Ti)Si compound. This complex compound exists in multiple morphologies, including spherical particles, thin and thick elongated platelets, and irregular-shaped particles. The size of these particles varies considerably, from large particles about 4 μm in length to very fine particles in the nano-scale. Similar observations were previously reported by Garza-Elizondo [5] for 354-type Al-Si-Cu-Mg alloys with different percentages of Ni and Zr additions. The presence of these particles is considered to be very beneficial to the mechanical performance at elevated temperatures because they are known to be thermally stable particles which resist coarsening and hence maintain acceptable values of the mechanical properties at elevated temperatures [10,41].

The limited variation in the elevated-temperature strength values of the alloys before and after stabilization treatment, seen in Figure 4a, can be understood in terms of some factors, including: the existence of the thermally stable Zr-containing dispersoids in the five alloys studied owing to the same Zr content in all alloys, the similar casting procedures followed in producing all test bars, and the same parameters used in the applied heat treatments.

**Figure 7:**
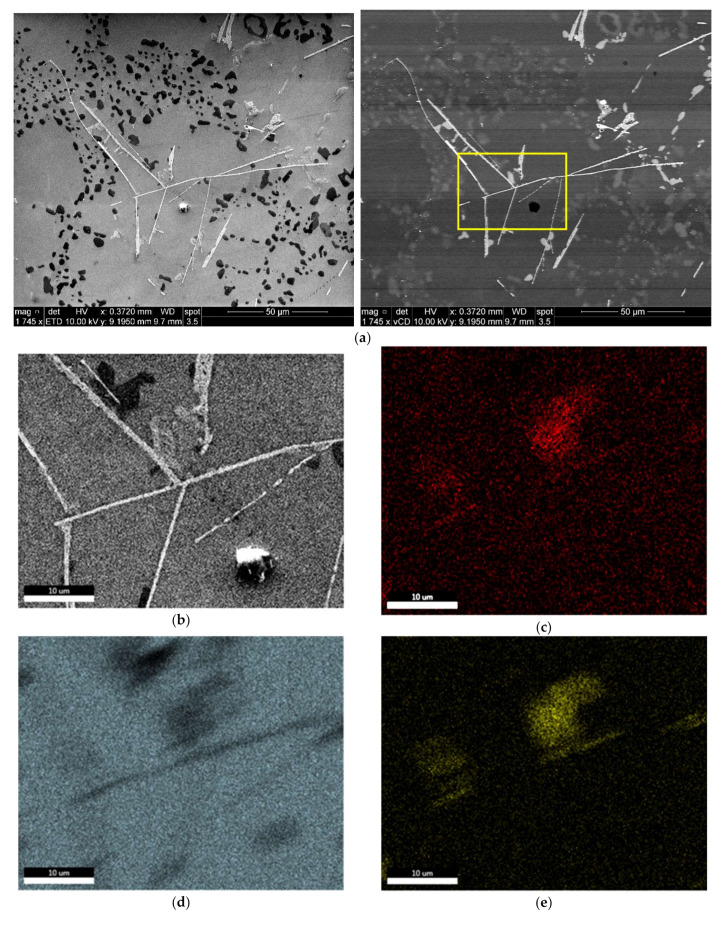

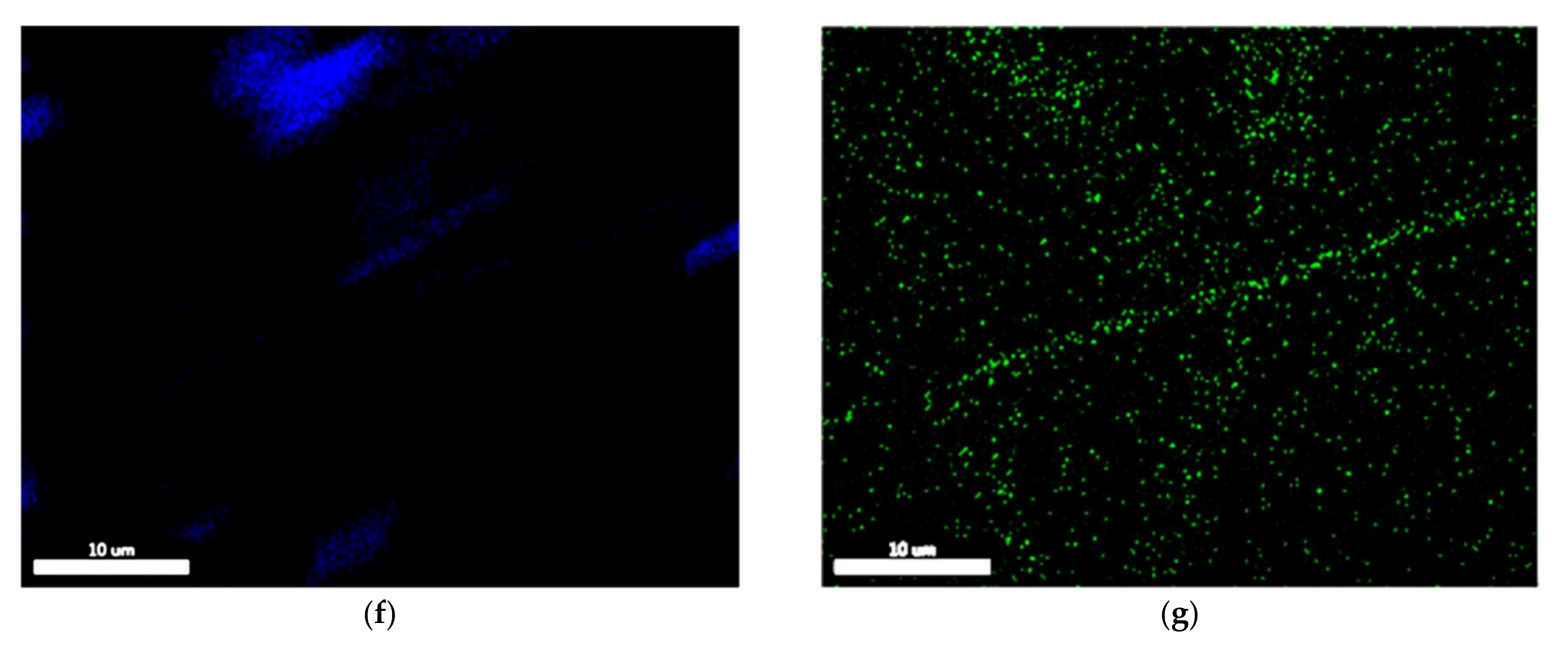
(**a**) Backscattered electron image of T6-treated M3S alloy after stabilization at 250 °C for 1 h; (**b**) higher magnification image of the inset in (**a**); (**c**–**g**) X-ray maps showing the distribution of elements in (**b**): (**c**) Cu, (**d**) Al, (**e**) Mg, (**f**) Si, (**g**) Mn.

The backscattered electron (BSE) image shown in Figure 7a exhibits some interesting features obtained from the T6-treated M3S alloy after stabilization for one hour at 250 °C. A high magnification image of the inset in (a) highlights the microstructural features observed more clearly, as shown in Figure 7b. The elemental distribution maps corresponding to the different elements present are also shown in Figure 7. The point of interest in this figure is the distribution of Mn, Figure 7g, which shows a faint reflection over the field of the image. This indicates that Mn might be distributed across the microstructure on a small scale that is possibly related to the formation of tiny Al_6_Mn particles [39,42], which are considered one of the main reasons for the improved mechanical performance of Mn-containing alloys. 

**Figure 8:**
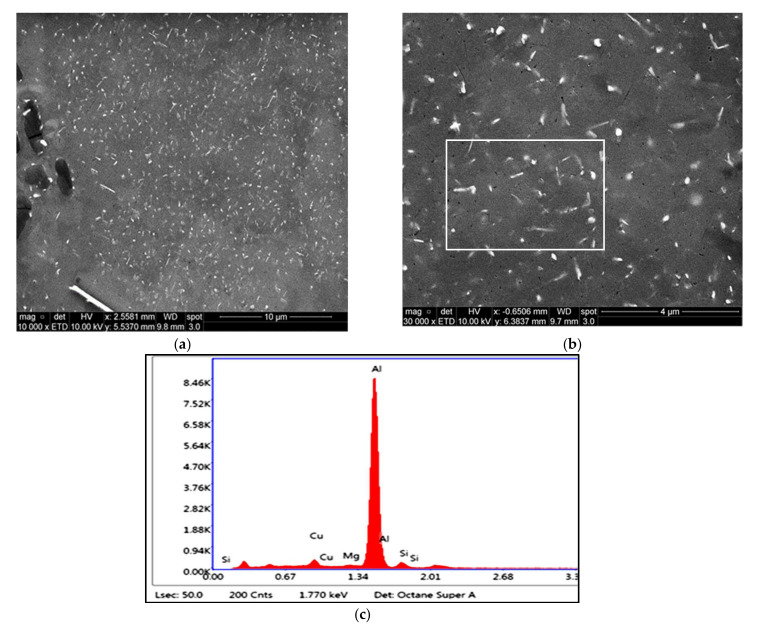
(**a**,**b**) Backscattered electron images at different magnifications showing the size and distribution of precipitates in the T6-treated M1S alloy after stabilization at 250 °C for 200 h; (**c**) EDS spectrum corresponding to the rod-like particles in white square in (**b**).

Figure 8a is a BSE image showing a general view of the precipitates in the microstructure of the T6-treated base alloy M1S after stabilization for 200 h at 250 °C and testing at 250 °C. A higher magnification BSE image for the same condition, shown in Figure 8b, reveal the distribution of the coarsened precipitates. The corresponding EDS spectrum, Figure 8c, of these precipitates shows reflections of Al and Cu, which is possibly due to the Al_2_Cu phase. Generally, the orientation of the rod-like Al_2_Cu particles is established to lie along the <110> family of directions [43]. Specifically for these coarsened particles of Al_2_Cu in Figure 8, they appear to be originally oriented along two perpendicular directions; in these images, however, these particles do not show a perfect perpendicularity, given that the sample examined was obtained from a tensile-tested bar.

**Figure 9:**
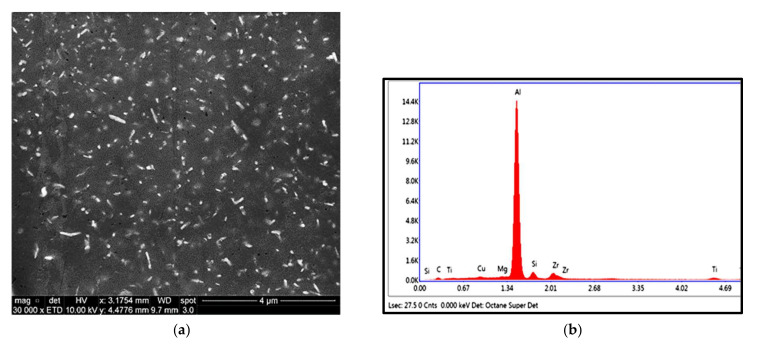
Backscattered electron image showing (**a**) bright, coarse, and fine dispersoids in T6-treated M2S alloy after stabilization at 250 °C for 200 h and testing at the same temperature; (**b**) EDS spectrum of bright particles in (**a**).

After the stabilization of the T6-treated alloy M2S for 200 h at 250 °C, the microstructure still contains a considerable amount of the very fine bright precipitates, as shown in Figure 9a. The corresponding EDS spectrum in Figure 9b reveals that these tiny precipitates are most likely the coarsening-resistant Zr-containing compounds. The presence of tiny Zr-containing precipitates can be easily observed in Figure 9a (i.e., after 200 h at 250 °C), which proves the thermal stability of such Zr-containing precipitates and hence their vital role in resisting alloy softening when employed in elevated-temperature applications.

**Figure 10:**
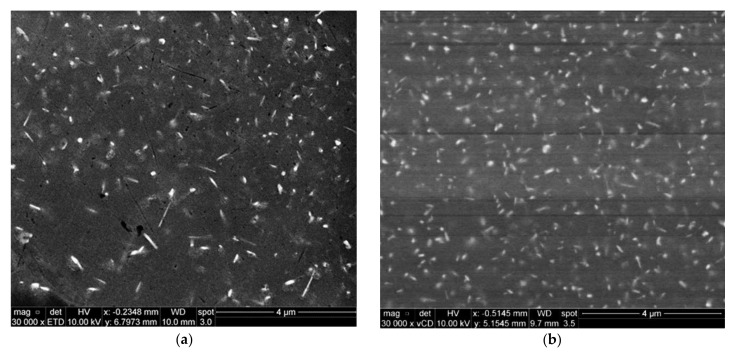
Backscattered electron images showing the density and distribution of the strengthening precipitates in T6-treated alloys after stabilization at 250 °C for 200 h: (**a**) M2S, and (**b**) M3S alloy (tested at 250 °C).

The high magnification BSE image presented in Figure 10a shows the microstructure of the same T6-treated alloy M2S stabilized for 200 h, highlighting the paucity of Al_2_Cu precipitates, attributable to the consumption of the Cu available for strengthening in forming other phases such as Al-Cu-Ni. On the other hand, the BSE image shown in Figure 10b demonstrates the fine distribution of the coarsened Al_2_Cu precipitates in the interdendritic regions in the microstructure of T6-treated M3S alloy under the same stabilization conditions (200 h at 250 °C).

**Figure 11:**
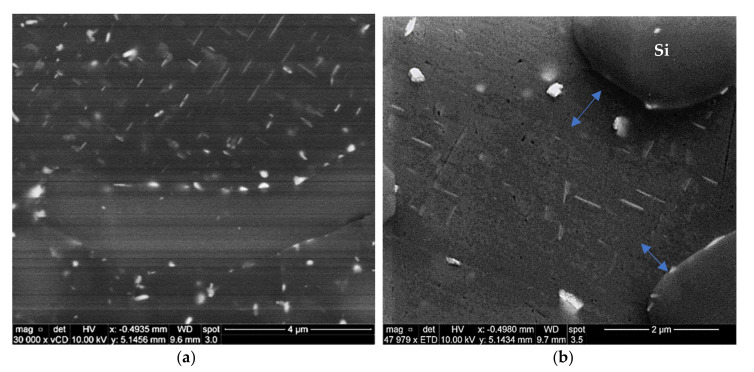
Backscattered electron images from T6-treated M2S alloy after stabilization at 250 °C for 200 h: (**a**) low magnification, (**b**) high magnification of (**a**) showing PFZs (blue arrows).

The BSE image of Figure 11 shows that the size of the precipitate free zones (PFZs) in the microstructure of the T6-treated M3S alloy after stabilization for 200 h at 250 °C is relatively small, taking into account the reduced number of precipitates. This is in keeping with the coarsening behavior, which increases the distances between neighboring precipitates, and contributes positively to the mechanical performance of the M3S alloy, as depicted in Figure 4a. 

**Figure 12:**
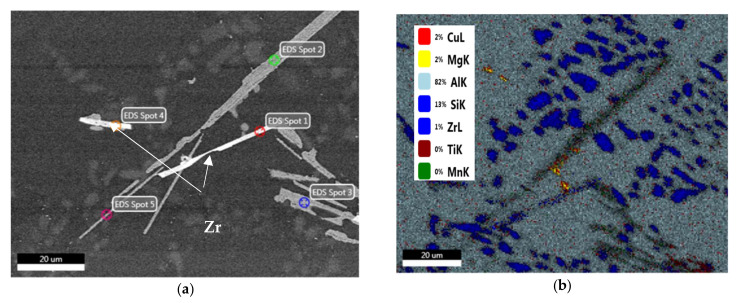

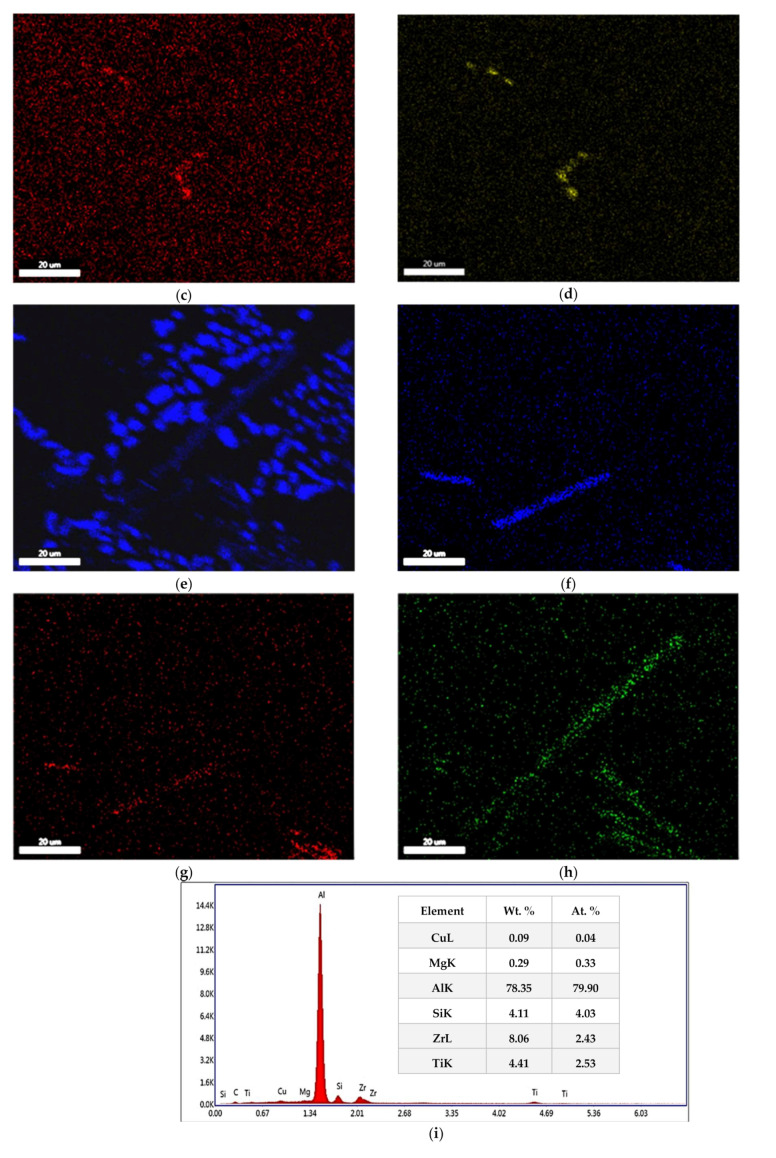

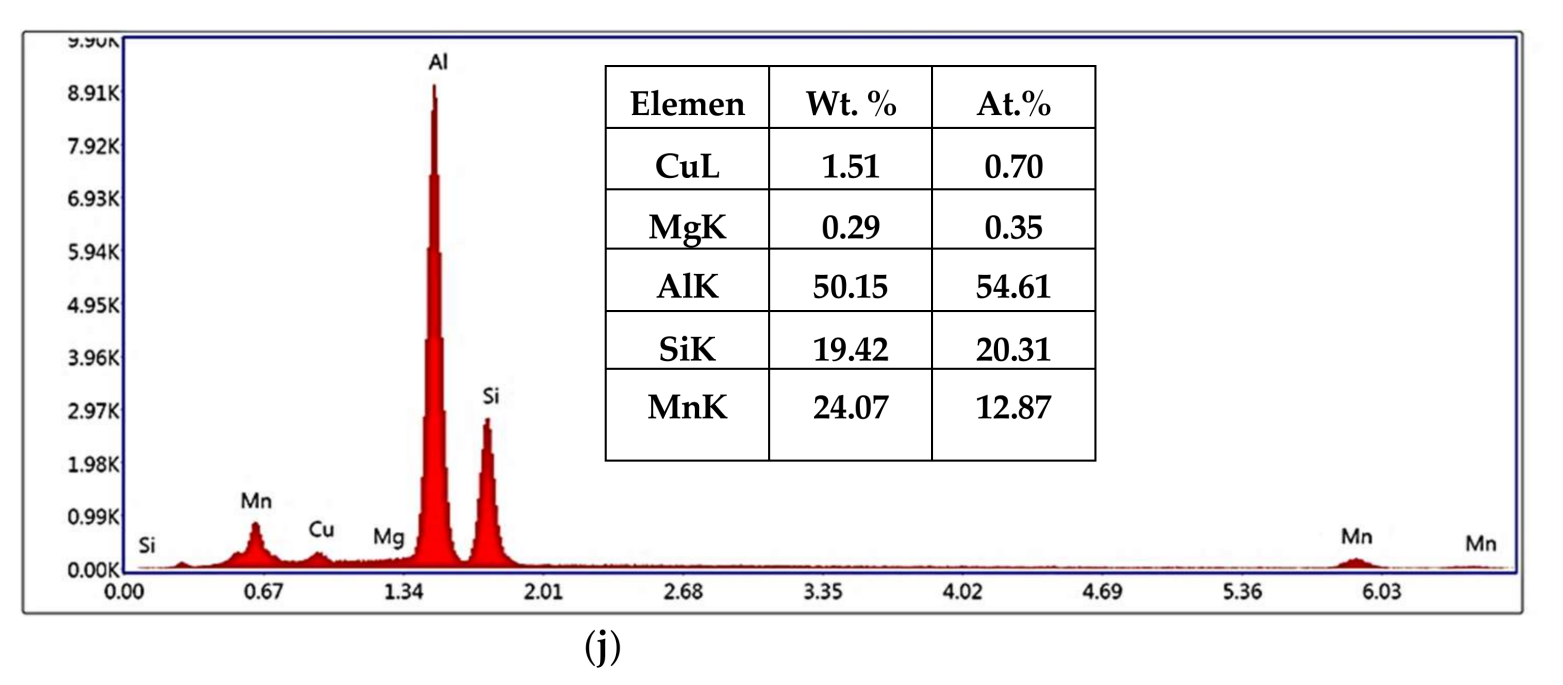
(**a**) Backscattered electron image of T6-treated M3S alloy after stabilization at 250 °C for 200 h; (**b**–**h**) corresponding X-ray maps showing distribution of elements in (**a**): (**b**) map of all elements, (**c**) Al, (**d**) Mg, (**e**) Si, (**f**) Zr, (**g**) Ti, (**h**) Mn; (**i**) EDS spectrum corresponding to spot #1 in (**a**); (**j**) EDS spectrum corresponding to spot #2 in (**a**).

The fine Al_6_Mn precipitates are considered to be responsible for the increased strength and ductility values of the M3S alloy at room temperature and at 250 °C, as well as before and after the stabilization treatment. Thus, by investigating the distribution of elements in the microstructure of the stabilized T6-treated alloy M3S shown in Figure 12, the distribution of Mn noted in Figure 12h is promising in supporting the formation of these fine precipitates. Additionally, the distribution of Zr, shown in Figure 12f, reveals that fine Zr-containing precipitates are likely to form as well. Therefore, improved mechanical performance at elevated temperatures is expected for alloy M3S. 

**Figure 13:**
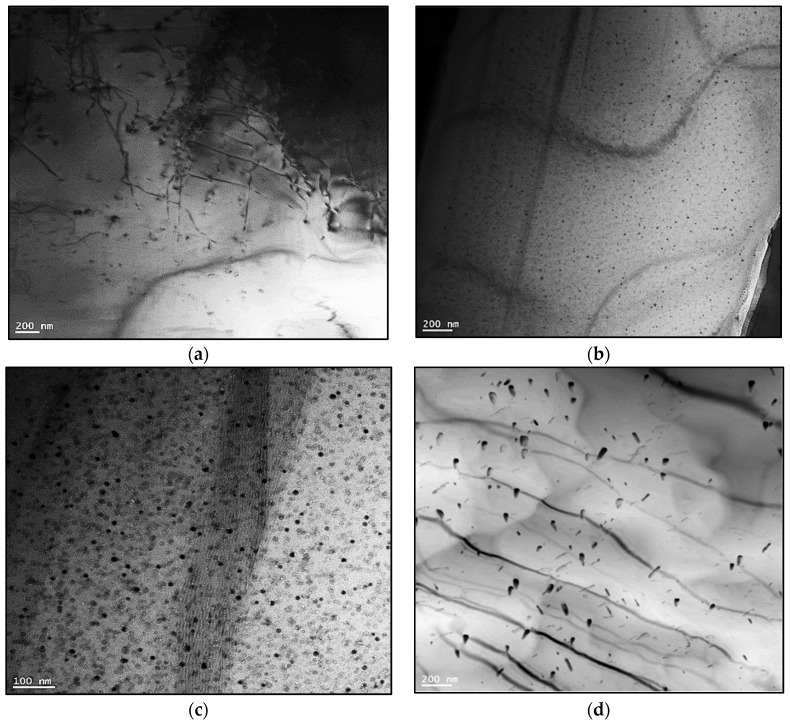
Bright field TEM images of precipitation during heat treatment of Sr-modified M4S alloy following (**a**) solution heat treatment, (**b**) T5 temper, (**c**) T6 + 8 h/250 °C, (**d**) T6 + 200 h/250 °C. Note the progress in the size and density of precipitated phase particles (Al_2_Cu) on going from one treatment to another.

**Figure 14:**
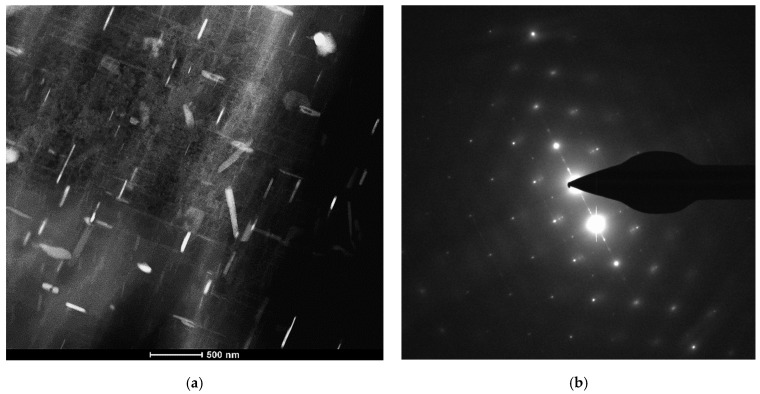
(**a**) High-angle annular dark-field image of alloy M1S in T6-treated condition (no stabilization treatment), and (**b**) the selected area electron diffraction (SAED) pattern.

To arrive at a better understanding of the effect of increasing the Ni content to 4% (i.e., M4S alloy) on the precipitation hardening, Figure 13 shows how the Ni-Cu interaction would lead to formation of Al-Ni-Cu intermetallics and hence reduce the effectiveness of Cu as a hardening agent compared to a Ni-free alloy [44], as in the case of M1S alloy shown in Figure 14 (T6 condition).

### 3.6. Fractography

**Figure 15:**
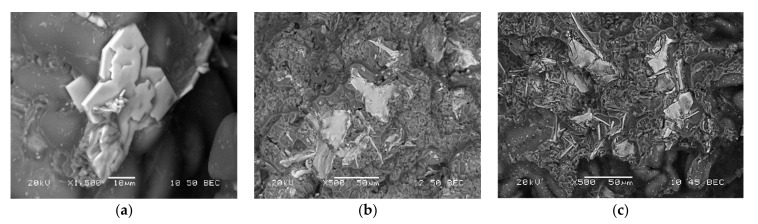
Fracture surface of M1S alloy in: (**a**) as cast, (**b**) SHT, and (**c**) T6 conditions.

In this section, the details of the fracture surfaces of M1S, M2S, and M3S alloys in the as cast, SHT, and T6 conditions will be briefly discussed using backscattered electron images. Considering M1S, Figure 15a shows the presence of a large Zr-rich particle having a star-like shape (mainly Al-Ti-Zr phase). As mentioned in Table 1, all alloys are containing about 0.3% Zr; thus, the precipitation of such coarse particles during the course of solidification is expected. Upon solution heat treatment (SHT) at 495 °C/5 h, some of these particles can still be seen on the fracture surface, as depicted in Figure 15b. With the increase in the alloy strength following artificial aging at 180 °C/8 h representing peak-aging [45], the fracture surface displayed in Figure 15c reveals fragmentation of the initial coarse particles.

**Figure 16:**
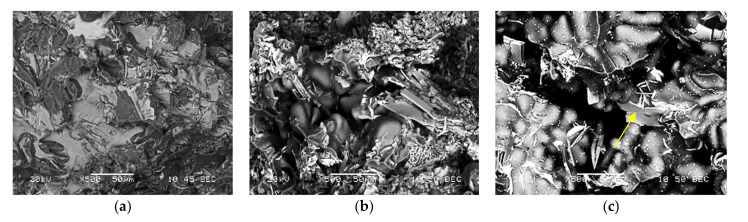
Fracture surface of M2S alloy in: (**a**) as cast, (**b**) SHT, and (**c**) T6 conditions.

**Figure 17:**
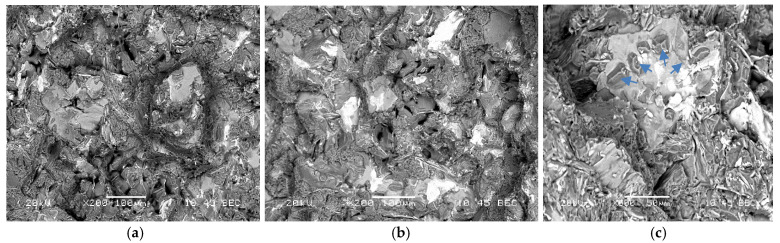
Fracture surface of M3S alloy in: (**a**) as cast, (**b**) SHT, and (**c**) T6 conditions. Blue arrows in (**c**) point to perforation of the AlSiMnFe-based phase.

Figure 16a represents the fracture surface of M2S alloy (containing about 2 wt.% Ni) in the as-cast condition. As can be seen, several large bright particles, mainly a mixture of Al-Ni, Al-Ni-Cu, and Al-Fe-Ni phases, are observed, which are almost insoluble in the aluminum matrix even after solution treatment, as depicted in Figure 16b. Aging at 180 °C resulted in intense precipitation of fine Al_2_Cu phase particles covering the surface of dendrites as shown in Figure 16c. The yellow arrow in Figure 16c points to a platelet of Al-Ni phase (image was viewed in a small pore). As in the case of M2S alloy, the fracture surface of M3S alloy exhibited the precipitation of several phases (mainly Al-Si-Mn-Fe and Al-Ti-Zr). Since these alloys were modified with about 200 ppm Sr, Figure 17c reveals perforation of the wide AlSiMnFe-based phase, as highlighted by the blue arrows.

## 4. Conclusions

This article addresses the concept of prolonged thermal exposure at 250 °C, or stabilization, and its effect on the mechanical performance of the alloys studied. The effects of prolonged thermal exposure at 250 °C for 100 and 200 h on the mechanical performance of the T5- and T6-treated alloys were examined, covering (i) ambient- and elevated-temperature tensile properties, (ii) ambient-temperature hardness values, and (iii) evolution of strengthening precipitates in the alloys studied, (iv) analysis of precipitation applying both SEM and STEM techniques. An analysis of the experimental data presented here led to the following conclusions.

1. Coarsening of the strengthening precipitates following prolonged exposure at 250 °C has a deleterious effect on the tensile strength and hardness values, thereby resulting in a significant increase in the ductility values.
2. The coarsening kinetics of the precipitates decay with time, due to the continuously increased distance between the precipitates with increase in the exposure time, causing deterioration in the mechanical performance after thermal exposure at 250 °C up to 100 h. Further thermal exposure up to 200 h does not reduce the strength and hardness values.
3. The strength values (UTS and YS) obtained at room temperature for the stabilized T5-treated conditions are comparable to, and in most alloys exceed, those of the stabilized T6-treated conditions.
4. In the case of elevated-temperature tensile testing, the T5 strength values in the stabilized conditions are always higher than those obtained for the stabilized T6 condition.
5. Addition of 0.75 wt.% Mn is competitive with the addition of 2 and 4 wt.% Ni with respect to the elevated- and ambient-temperature strength values, respectively, with the Mn-containing alloy providing the advantage of higher ductility values.
6. Precipitation of different strengthening/intermetallic phases identified from the TEM investigations include Al_2_Cu, Al-Ni-Cu, Al-Ni, Al-Si-Mn-Fe, Al-Ti-Zr phases, and fine Zr-rich and Al_6_Mn precipitates. The phases present and their evolution with the type and duration of the heat treatment determine the alloy properties.

## Figures and Tables

**Table 1:** Chemistry of the alloys studied.

Chemical Analysis (wt.%)											
Alloy Description		Elements									
Type	Code	Si	Cu	Mg	Fe	Ti	Zr	Ni	Mn	Sr	Al
354	M1S	8.5	1.76	0.55	0.12	0.2	0.32	<0.1	0.01	0.02	Bal.
	M2S	8.4	1.7	0.60	0.14	0.21	0.33	1.9	0.01	0.02	Bal.
	M3S	8.6	1.8	0.55	0.11	0.20	0.33	<0.1	0.75	0.02	Bal.
	M4S	8.6	1.8	0.67	0.12	0.22	0.29	4.0	0.01	0.02	Bal.
	M5S	8.6	1.8	0.60	0.15	0.20	0.29	1.9	0.76	0.02	Bal.

**Table 2:** Heat treatment procedures and parameters applied to the alloys investigated.

Heat Treatment Procedures and Parameters			
Heat Treatment	Solution Treatment	Quenching	Aging
T5	NA	NA	8 h @ 180 °C
T6	5 h @ 495 °C	Warm water (60 °C)	8 h @ 180 °C

**Table 3:** Volume fractions (%) of undissolved intermetallic compounds in the matrix of as-quenched alloys following solution heat treatment (SHT).

Volume Fraction (%)		Alloy Code				
		M1S	M2S	M3S	M4S	M5S
SHT	Average	1.11	5.54	3.64	9.60	7.68
	SD	0.28	0.61	0.16	0.65	0.52

**Figure A1:**
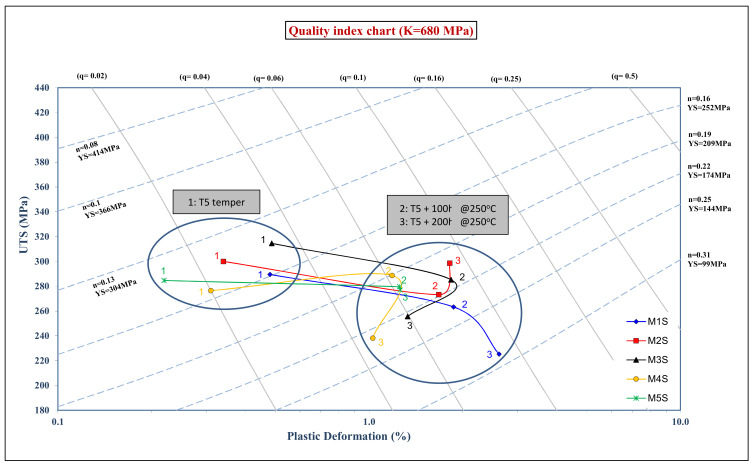
Cáceres quality chart representing the relation between UTS and percent plastic deformation of alloys M1S through M5S in the T5 condition before and after stabilization at 250 °C for 100 and 200 h (testing at ambient temperature).

**Figure A2:**
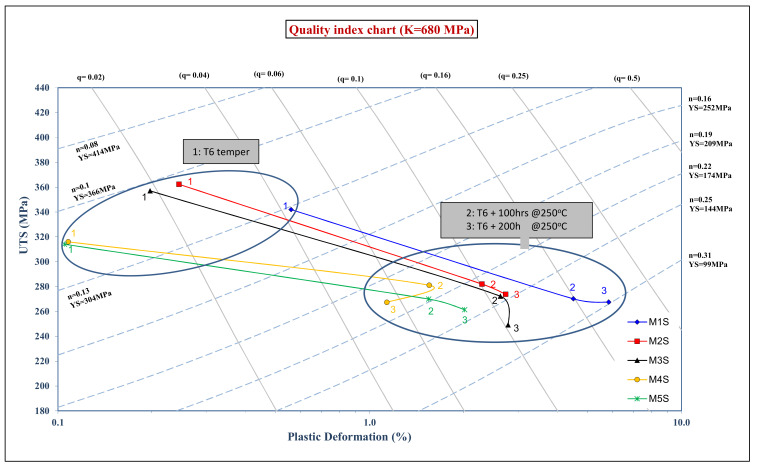
Cáceres quality chart representing the relation between UTS and percent plastic deformation of alloys M1S through M5S in the T6 condition before and after stabilization at 250 °C for 100 and 200 h (testing at ambient temperature).

**Figure A3:**
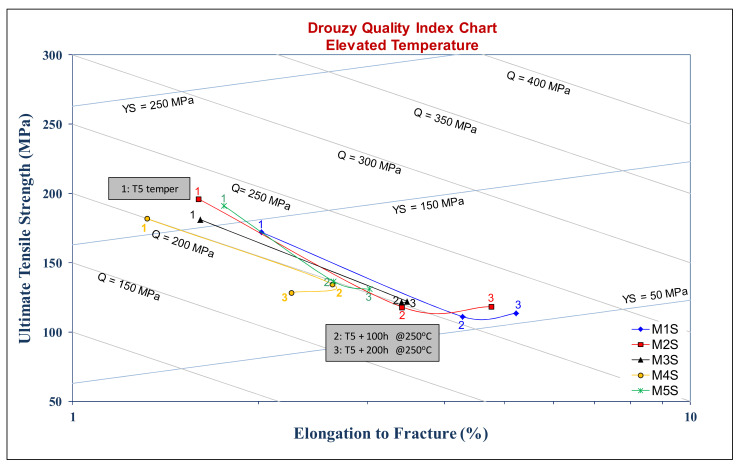
Drouzy quality chart representing the relation between the UTS and the percent elongation to fracture of alloys M1S through M5S in the T5-treated condition, before and after stabilization at 250 °C for 100 and 200 h (testing at 250 °C).

**Figure A4:**
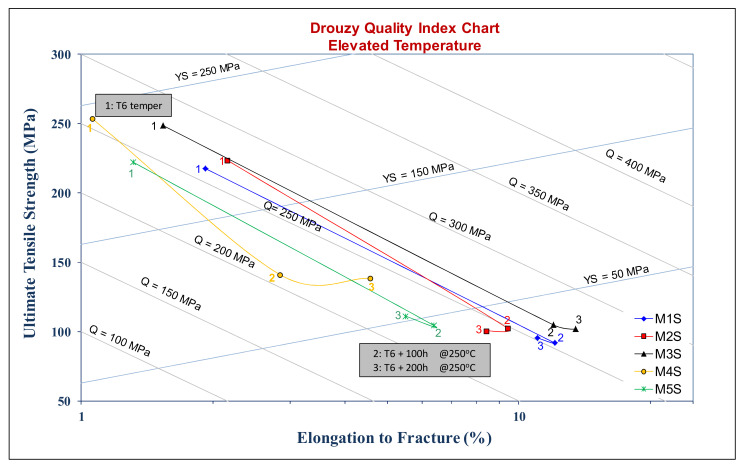
Drouzy quality chart representing the relation between the UTS and the percent elongation to fracture of alloys M1S through M5S in the T6-treated condition, before and after stabilization at 250 °C for 100 and 200 h (testing at 250 °C).

## Data Availability

Data is available upon request.

## Appendix A. Quality Index

The concept of the quality index (*Q*) was originally developed in 1980 by Drouzy et al. [17], followed by further improvements proposed by researchers such as Cáceres [18]. Quality charts constructed from *Q* values and the tensile properties of specific alloys are useful in selecting the optimum conditions that will provide superior tensile properties and optimum quality. The quality index value (*Q*) is intrinsically related to the quality of the castings, which is susceptible to improvement through adequate control of impurity elements, casting defects, modification, solution heat treatment, and solidification conditions. The probable yield strength (YS) depends mainly on the presence of hardening elements such as Mg and Cu, and on the age-hardening conditions applied to the castings [17,18,19]. Drouzy et al. [17] proposed the new concept of quality index (*Q*) to express the performance of cast aluminum alloys, where the authors related the quality index of these alloys to their mechanical properties, namely, the ultimate tensile strength and percentage elongation to fracture. The curvilinear contours that may appear in the quality charts can be attributed to variation in the aging conditions [20,21], which is mainly observed in Cu-containing Al alloys. Thus, it will be important to determine the behavior of the strength-ductility relationship as the material undergoes the aging process. The quality index was empirically developed using the following equation:

(A1) $$Q=S_{UTS}+d\log\left(e_{f}\right)$$

where *Q* is the quality index (MPa); *S_UTS_* refers to the ultimate tensile strength (MPa), *e_f_* refers to the percentage elongation to fracture, and *d* is a material constant equal to 150 MPa.

The probable yield strength (*S_p(ys)_*) was identified by the following formula:

(A2) $$S_{p\left(ys\right)}=aS_{UTS}-b\log\left(e_{f}\right)+C$$

where coefficients *a*, *b*, and *c* were quantified as 1, 60 MPa, and −13 MPa, respectively.

Cáceres developed a theoretical model which was capable of describing the physical significance of the quality index [18,21,22]. He developed his model on the assumption that the material undergoes a plastic deformation that may be described by the Holloman equation:

(A3) $$\sigma =K\varepsilon^{n}$$

where *σ* is the true stress (MPa), ε is the true plastic strain, n is the strain-hardening exponent, and K is the strength coefficient (MPa).

Cáceres proposed the term relative quality index (*q*) based on the assumption that necking will start when *e_u_* = *ε_u_* = *n*, where *e_u_* and *ε_u_* represent the engineering and true strain, respectively, at the onset of necking. The relative quality index values may be represented by iso-lines describing the ratio between the percent engineering strain to fracture, *e_f_*, and the critical engineering strain, *e_u_*, so that the relative quality index may be expressed by [18,22,23,24,25,26,27,28,29,30,31]:

(A4) $$q=\frac{e_{f}}{e_{u}}≅\;\frac{e_{f}}{n}$$

After mathematical manipulations, Cáceres developed the following two equations which were used to generate the iso-flow lines and iso-q lines, respectively, in the quality chart proposed by Cáceres:

(A5) $$S=K{\left[\ln\left(1+e\right)\right]}^{n}\;exp^{-\ln\left(1+e\right)}≅Ke^{n}\;exp^{-e}$$

(A6) $$S=K\;e^{\frac{e}{q}}\;exp^{-e}$$

where *S* is the engineering stress.

The Cáceres quality index can be calculated from the tensile test results using the following equation [30]:

(A7) $$Q_{c}=S_{UTS}+0.4K\;\log\left(e_{f}\right)$$

Equations (A1) and (A2) are used to generate iso-Q lines and iso-YS lines, respectively, in Drouzy’s quality charts, whereas Equations (A5) and (A6) are used to generate the iso-flow lines and iso-q lines, respectively, in the quality chart proposed by Cáceres.

1- Quality Index at Ambient Temperature:

Cáceres quality charts for the stabilized T5- and T6-treated conditions of the alloys studied are shown in Figure A1 and Figure A2, respectively. These charts show variations in the quality of the alloys studied based on the tensile test data obtained in the stabilized T5- and T6-treated conditions at ambient temperature. The two figures reveal that the quality values of the alloys studied improved remarkably after the prolonged elevated-temperature exposure at 250 °C owing to the highly improved plastic deformation values, despite the reduced strength values.

Regardless of the differences in quality values of the alloys M1S through M3S, it is obvious that these three alloys display the best quality values rather than alloys M4S and M5S. For the base alloy M1S, stabilization of the T6-treated condition at 250 °C for 100 and 200 h, as shown in Figure A2, produces the best quality index because of its significantly improved ductility. The quality indices of alloys M2S and M3S in the stabilized T6-treated conditions, i.e., 100 and 200 h, exhibit more or less the same values. However, for the stabilized T5-treated conditions of alloys M1S through M3S, the quality indices obtained for these conditions do not vary considerably, except for the 200 h stabilized T5-treated condition of the 0.75% Mn-containing M3S alloy.

2- Quality Index at Elevated Temperature:

Quality index charts, according to the model developed by Drouzy et al. [17], are shown in Figure A3 and Figure A4, respectively, for stabilized T5- and T6-treated conditions of the alloys studied. Generally, the quality index values for stabilized T6-treated conditions are higher than those for the stabilized T5-treated conditions and may be attributed to the improved ductility values of the stabilized T6-treated alloys, owing to the high proportion of coarsened strengthening precipitates, even though the UTS values are higher for the stabilized T5-treated alloys.

The prolonged thermal exposure produces balanced variations in the ultimate tensile strength and ductility values of the alloys studied, i.e., increased strength values concomitant to reduced ductility values, and vice versa. This balanced variation in UTS and ductility values results in slight discrepancies in the quality index values for the various alloy/conditions studied. Another observation from Figure A3 regarding the stabilized T5-treated conditions is that the quality indices are so close for all the investigated alloys after applying the stabilization treatment. In contrast, Figure A4 shows a clear sorting of the high quality index values of alloys M1S, M2S, and M3S, and the reduced quality index values of alloys M4S and M5S. This observation may be understood in terms of the reported low ductility values of alloys M4S and M5S in spite of their improved elevated-temperature ultimate strength obtained after the stabilization treatment.
